# Once Bitten, Twice Shy: On the Transient Nature of Congruency Sequence Effects

**DOI:** 10.3389/fpsyg.2012.00264

**Published:** 2012-07-26

**Authors:** Wery P. M. van den Wildenberg, K. Richard Ridderinkhof, Scott A. Wylie

**Affiliations:** ^1^Amsterdam Center for the Study of Adaptive Control in Brain and Behavior, Department of Psychology, University of AmsterdamAmsterdam, Netherlands; ^2^Cognitive Science Center Amsterdam, University of AmsterdamAmsterdam, Netherlands; ^3^Department of Neurology, Vanderbilt University Medical CenterNashville, TN, USA

Life is ambiguous at times, presenting conflicting situations and conflicting response tendencies. For example, when confronted with a jinxing soccer opponent, you might easily be fooled by a skillful leg feint and fail to react to the direction of the ball. Here, your goal-directed action – no pun intended – is executed by resisting the impulse to react to the distracting jinx maneuver and instead reacting to the ball.

To explore adaptive cognitive control to resolve situations of interference and response conflict, as in the soccer example, few tasks in the toolkit have been as illustrious as the Stroop task (Stroop, 1935) named after its creator, John Ridley Stroop (1897–1973). Instructed to name the font color of a word, responses are typically fast and accurate if there is no conflict (e.g., say “blue” to the *congruent* stimulus 
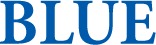
). Answers to *incongruent* stimuli (e.g., 
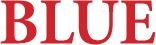
) generally take longer and are less accurate because of the strong tendency to read the word and mistakenly say “blue” instead of “red.” This performance difference is called the *congruency effect* and its magnitude is widely used to study how well individuals can resist interference.

Particularly interesting is the discovery by Gratton et al. (1992) that the magnitude of interference depends on recent history. Let's again consider a soccer example. After just being jinxed, you will make sure to avoid being fooled again by distracting feints on the next encounter and direct your reaction to the ball (i.e., “once bitten, twice shy”). This illustrates that after facing conflict, people can adapt quickly to counteract the performance reducing effects of future conflict. In the Stroop task and similar conflict tasks, these adaptive sequential effects are revealed by two patterns: (i) faster incongruent responses and slower congruent responses after incongruent trials as well as (ii) faster congruent responses and slower incongruent responses after congruent trials (Figure 1A). Thus, adaptation not only occurs after conflict *per se*, but depends on the congruency of the previous trial: (i) if the going gets tough, the tough get going (control effort is increased, the gates are closed, after an incongruent trial), and (ii) if the going gets easy, the gates are opened (control is relaxed after a congruent trial).

**Figure 1:**
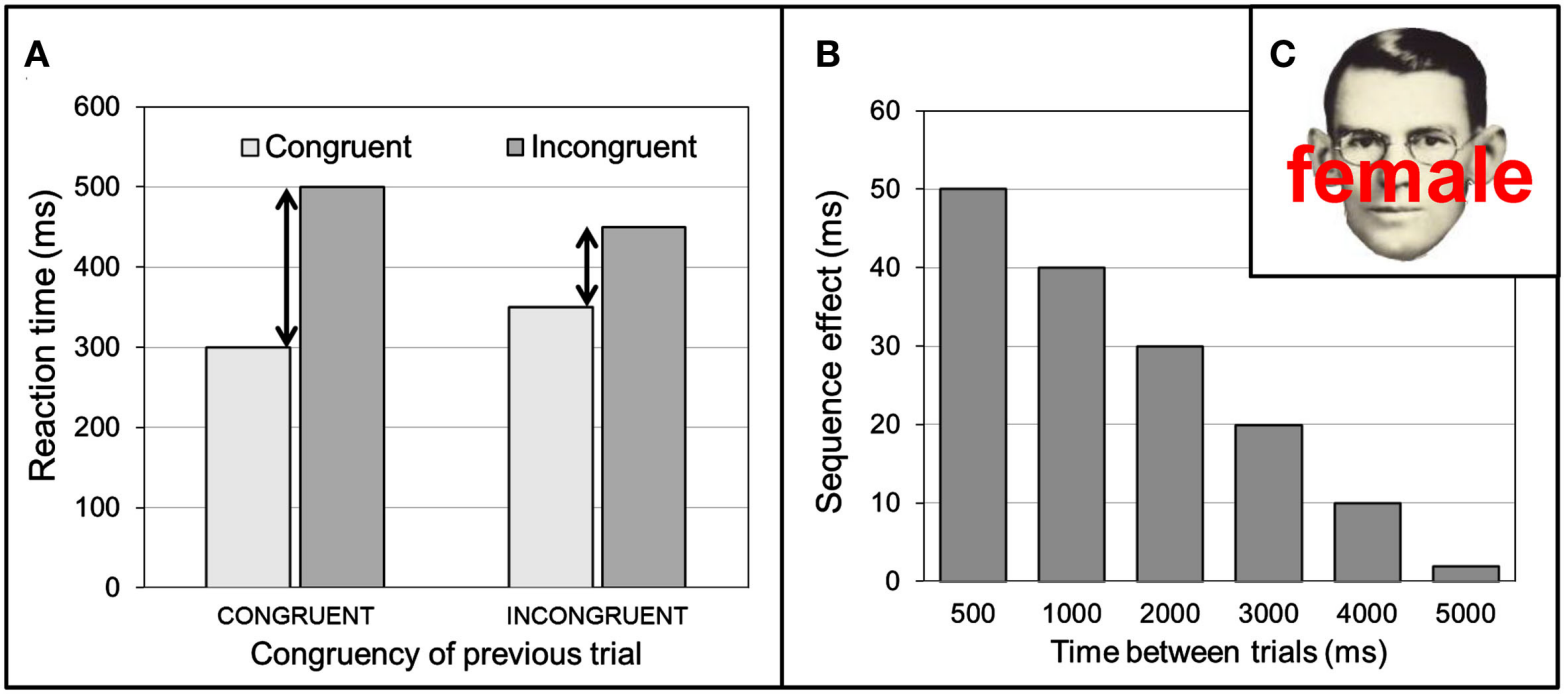
**(A)** The interference effect, indicated by the size of the black arrows, is reduced after an incongruent trial compared to after a congruent trial. **(B)** The congruency sequence effect [CSE; the difference in size between the two arrows in **(A)**] reduces as a function of time. **(C)** Example of an incongruent Stroop Face-Word stimulus. Note: **(A,B)** depict fictitious data.

Botvinick et al. (2001) used the term *Gratton effect* to describe this pattern, but because they offered an influential interpretation of the effect in terms of conflict adaptation, the phenomenon has widely been coined the *conflict adaptation effect*. As a more theory-neutral term, we adopt the operational term *congruency sequence effect* (CSE), following Egner et al. (2010). In their insightful paper published in *Frontiers in Psychology*, Egner and colleagues addressed the unresolved issue of the time-course of CSE. To determine whether CSE is short-lived or persists for several seconds, they systematically varied the interval between subsequent face-word Stroop stimuli from 500 to 7,000 ms (see Figure 1C). As expected, responses to incongruent face-word stimuli were significantly slower than responses to congruent face-word stimuli, reflecting the additional time to resolve the interference. Second, this congruency effect was modulated by the congruency of the preceding trial. That is, following congruent trials, interference was quite large, but after incongruent trials the interference effect diminished to just a few ms (Figure 1A). Most important is the demonstration that CSE steadily diminished with time, despite an exponential increase in the likelihood of stimulus appearance with increasing interval duration (i.e., an exponential hazard function), making their finding all the more non-trivial. Disappearing within 4,000 ms, adaptive effects appear to be rather short-lived (Figure 1B).

Several accounts have been offered to explain CSE, including two influential views that emphasize the role of top-down cognitive control processes. Gratton et al. (1992) emphasized the role of expectancy or anticipation as preparing for expedient processing of future conflict. On this account, one would predict that the more time passes after a conflict trial, the better prepared one should be to handle further conflict, and hence CSE should increase. This prediction is clearly refuted by the Egner et al. (2010) findings. By contrast, Botvinick et al. (2001) attributed CSE to the role of attentional control engaged to reduce the detrimental impact of task-irrelevant stimulus processing in the event of future instances of conflict. This view predicts that top-down attentional control is stronger immediately following instances of conflict, but likely dissipates as time elapses between trials, consistent with Egner et al.’s findings of CSE diminishing over time. It should be noted, however, that at least part of the CSE pertains to action control rather than attentional control (van den Wildenberg et al., 2010). For instance, incongruent trials are followed by reduced impulse capture (reflected by fewer fast errors) and augmented inhibitory control (reduced interference in the slower portions of the RT distribution; Wylie et al., 2010).

In sum, Egner et al. (2010) convincingly show that adaptive attentional control is rather transient, a finding with important implications. If CSE reflects proactive control (Gratton et al., 1992), then this form of control is rather short-lived, which would be bad news for the soccer player intent on not being jinxed on the next encounter. Instead, between-trial CSE may be viewed as manifestations of residual adaptation effects from within-trial conflict adaptation (Verguts and Notebaert, 2009). This would fit with its short-lived nature. It remains to be determined if this short breath works against or benefits goal-directed behavior. When it's difficult to predict the nature of the upcoming encounter, it may in fact be beneficial to cast off residual after-effects in order to confront the next trial in a relatively neutral state, being equally prepared for an upcoming congruent as for an incongruent trial. After all, body and leg motion often *is* predictive of where the opponent will play the ball, and responding to such information may in general be advantageous enough to risk the incidental jinx.

## References

[B1] Botvinick M. M. Braver T. S. Barch D. M. Carter C. S. Cohen J. D. (2001). Conflict monitoring and cognitive control. Psychol. Rev. 108, 624–652 10.1037/0033-295X.108.3.624 11488380

[B2] Egner T. Ely S. Grinband J. (2010). Going, going, gone: characterizing the time-course of congruency sequence effects. Front. Psychol. 1:154 10.3389/fpsyg.2010.00154 21833220 PMC3153769

[B3] Gratton G. Coles M. G. Donchin E. (1992). Optimizing the use of information: strategic control of activation of responses. J. Exp. Psychol. Gen. 121, 480–506 10.1037/0096-3445.121.4.480 1431740

[B4] Stroop J. R. (1935). Studies of interference in serial verbal reactions. J. Exp. Psychol. 18, 643–662 10.1037/h0054651

[B5] van den Wildenberg W. P. M. Wylie S. A. Forstmann B. U. Burle B. Hasbroucq T. Ridderinkhof K. R. (2010). To head or to heed? Beyond the surface of selective action inhibition: a review. Front. Hum. Neurosci. 4:222 10.3389/fnhum.2010.00222 21179583 PMC3004391

[B6] Verguts T. Notebaert W. (2009). Adaptation by binding: a learning account of cognitive control. Trends Cogn. Sci. (Regul. Ed.) 13, 252–257 10.1016/j.tics.2009.02.007 19428288

[B7] Wylie S. A. Ridderinkhof K. R. Bashore T. R. van den Wildenberg W. P. M. (2010). The effect of Parkinson's disease on the dynamics of online and proactive cognitive control during action selection. J. Cogn. Neurosci. 22, 2058–2073 10.1162/jocn.2009.21326 19702465 PMC2923490

